# Assessing Functional Changes With the Integration of Wrist Flexion Into a Myoelectric Prosthesis

**DOI:** 10.1109/TNSRE.2025.3646472

**Published:** 2026

**Authors:** Laura A. Miller, Kristi L. Turner, Kevin Brenner, Levi J. Hargrove

**Affiliations:** Center for Bionic Medicine, Shirley Ryan AbilityLab, Chicago, IL 60611 USA; Department of Physical Medicine and Rehabilitation, Northwestern University, Chicago, IL 60611 USA; Center for Bionic Medicine, Shirley Ryan AbilityLab, Chicago, IL 60611 USA; Center for Bionic Medicine, Shirley Ryan AbilityLab, Chicago, IL 60611 USA; Center for Bionic Medicine, Shirley Ryan AbilityLab, Chicago, IL 60611 USA; Department of Biomedical Engineering and the Department of Physical Medicine and Rehabilitation, Northwestern University, Chicago, IL 60611 USA

**Keywords:** Degrees of freedom, myoelectric devices, pattern recognition, performance evaluation, prosthetics

## Abstract

This study investigates functional performance using a two-degree-of-freedom (2DOF) prosthetic wrist compared to a single-degree-of-freedom (1DOF) wrist in individuals with transradial (below-elbow) amputation. Five participants were fitted with a custom-designed 2DOF prosthetic wrist system integrated with an Ottobock Transcarpal hand and operated via a pattern recognition-based myoelectric control interface. Participants completed two test conditions: one using wrist rotation alone (1DOF, NoWF), and another using wrist rotation combined with wrist flexion and extension (2DOF, WF). A battery of standardized functional assessments was used to evaluate performance in both conditions, including the Southampton Hand Assessment Procedure (SHAP), Box and Blocks Test (BBT), Jebsen-Taylor Hand Function Test (JTHFT), Activity Measure for Upper Limb Amputees (AM-ULA), Clothespin Relocation Task (CRT), and the Assessment of Capacity for Myoelectric Control (ACMC). Across all outcome measures, no statistically significant differences were found between the 1DOF and 2DOF conditions. While the lack of measurable improvement may reflect the influence of factors inherent to the 2DOF design, such as its greater length, added mass compared to 1DOF wrists, or increased control complexity, the results nonetheless indicate that the addition of a second wrist degree of freedom did not compromise functional performance. These findings suggest that more complex multi-DOF systems can be implemented without detriment to user function, an encouraging result for the continued development of advanced upper-limb prosthetic technologies.

## I. Introduction

Upper-limb loss at the transradial level, below the elbow but above the wrist, is the most common type of major upper-limb amputation [1] and significantly impacts independence in activities of daily living such as feeding, dressing, and hygiene [2]. Most transradial amputations occur in young, active individuals, often as a result of trauma, and present a unique clinical challenge: restoring hand and wrist function while maintaining a lightweight, durable, and intuitive prosthetic system. This is often achieved using a myoelectric prosthesis that uses control information extracted from electromyographic (EMG) signals measured from remaining residual limb muscles [3].

Over the past two decades, over a dozen new multi-articulating hands have become commercially available [4]. These devices share some design characteristics, but have several differentiating factors such as their durability, size, speed, grip force. There have also been enhancements in control approaches including the commercial release of pattern recognition-based control approaches. Despite advances in multi-articulating prosthetic hands and pattern recognition–based control, commercial and clinical changes in wrist actuation have seen comparatively little innovation.

The wrist plays a pivotal role in positioning the hand for functional grasp, midline access, and stable manipulation of objects. Its primary degrees of freedom, pronation/supination (rotation), flexion/extension, and ulnar/radial deviation, are essential for natural arm use and reducing compensatory strategies involving the shoulder and trunk [5]. Passive prosthetic wrists, which allow manual repositioning and locking, provide both rotation and flexion/extension, but lack dynamic responsiveness. In contrast, most commercially available powered wrists provide only active rotation, despite longstanding user demand for powered flexion/extension to improve function and ease of use [6].

This stagnation in commercial wrist innovation is largely due to engineering constraints. Adding powered degrees of freedom increases device complexity, weight, and length—factors known to contribute to prosthesis abandonment. Moreover, the integration of intuitive control strategies becomes more challenging as the number of controllable joints increases. However, emerging technologies in miniature actuators, embedded systems, and machine learning–based control offer new opportunities to address these limitations [7], [8], [9].

Recent research in subjects without limb loss or absence using orthotic constraints has shown that when hand dexterity is limited, the addition of two wrist degrees of freedom, rotation and flexion/extension, can restore performance levels comparable to those of an intact hand with reduced wrist mobility. This highlights the potential impact of restoring wrist motion in myoelectric prostheses. Nonetheless, the true functional benefit of powered wrist flexion/extension remains understudied in individuals with amputation using clinically viable devices and control systems.

A critical consideration in evaluating prosthetic innovation is the method of assessment. While many prior studies exploring the functional value of wrist flexion/extension have relied on offline analyses [5], [10], virtual control [11], or experiments conducted with able-bodied individuals, these approaches may not fully capture the real-world challenges faced by individuals with limb difference. Testing in physically instantiated systems, particularly with limb-different users, is essential for understanding the true clinical impact of new prosthetic technologies, particularly as the advance toward higher technology readiness levels. Standardized outcome measures, combined with real-time control in realistic tasks, provide a more valid and ecologically relevant evaluation of prosthetic function. Several functional outcomes may be used to assess how a powered wrist DOF may change function, including those recommended by the Upper Limb Prosthetic Outcome Measures working group [12].

The present study investigates whether adding powered wrist flexion/extension to a pattern recognition–controlled transradial prosthesis, already equipped with a powered wrist rotator and a single-DOF terminal device, results in improved functional performance. By focusing on individuals with transradial amputations and leveraging embedded pattern recognition systems for intuitive control, we evaluate the clinical relevance of restoring a second wrist DOF using standardized outcome measures.

## II. Methods

### A. Participants.

Five adults with unilateral transradial limb loss (mean age at the time of enrollment: 37.8 years; range: 28–55) were recruited. All participants were male, had limb loss secondary to a traumatic injury, and had prior experience with myoelectric prosthetic use, though only three used such devices regularly. Demographic data, including prosthetic side, presence of targeted muscle reinnervation (TMR), and home prosthesis type, are recorded in Table I. Approval of all ethical and experimental procedures and protocols was granted by the Northwestern University IRB under Protocol STU00206531 approved 18 January 2018 and the study was registered with Clinical Trials (NCT04069793). All participants provided informed consent.

### B. Wrist Design.

**Fig. 1.**
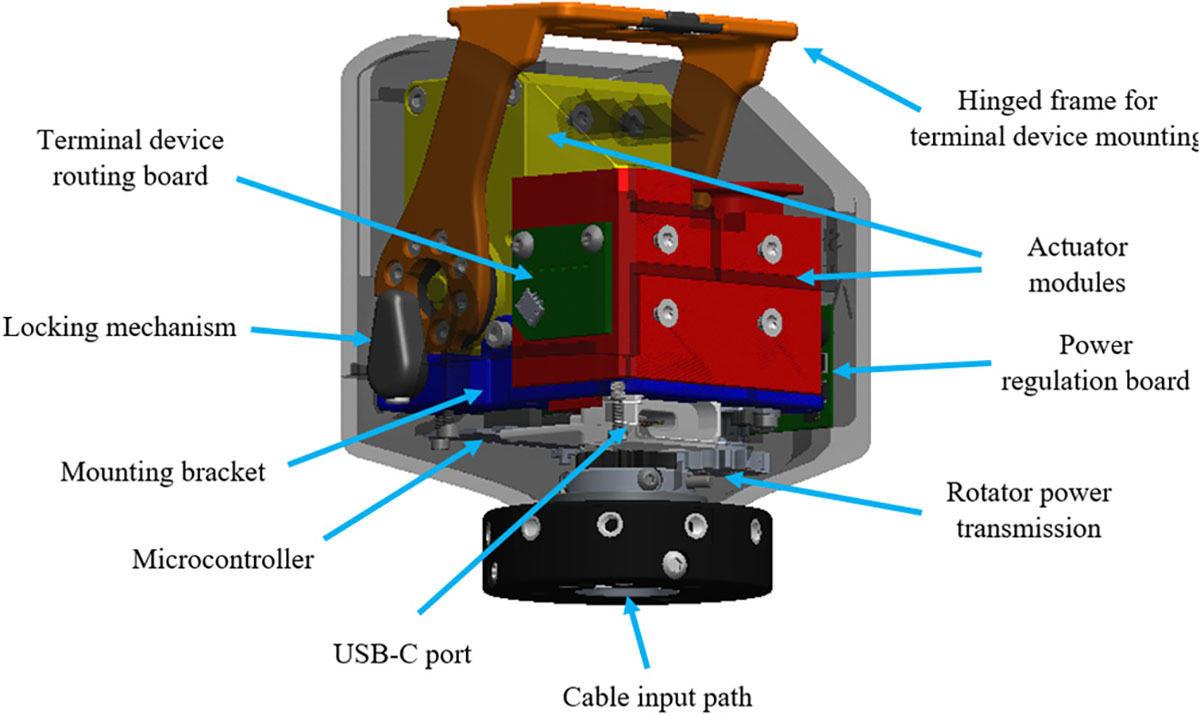
CAD model of the custom-designed 2-DOF prosthetic wrist identifying the main features and construction.

A custom 2-DOF wrist was fabricated with the joint goals of 1) minimizing overall build length, and 2) reducing total mass (Fig 1). To achieve this, two off-the-shelf Dynamixel actuator modules (XM430-W210-R) were selected for rotation and flexion/extension. These modules contain DC motors, motor controllers/drivers, feedback sensing, and compact gear reduction. Position and current data were read in from the motors, and desired velocity was written to the motors. This information was communicated over UART data lines between the actuator modules and an off-the-shelf microcontroller (Pyboard D-Series SF6W). The actuator modules each had a stall torque of 3.0 Nm and a no load speed of 77 rpm. Since there was no gear reduction in the transmission for either DOF, these were the theoretical values that each joint could achieve.

Additional features included 1) a custom designed outer frame, 2) a custom designed mounting bracket for each actuator module and the internal electronics, 3) power transmission for the rotator in order to align the wrist with the central axis of the forearm, 4) a locking mechanism for the flexor, and 5) a custom designed hinge frame used to mount the terminal device. Physical hard stops were built in for both degrees of freedom in order to ensure safety for the user and robustness for the device. The overall length and mass were calculated as 94.2 mm and 342 g, respectively (compared to a commercial Motion Control wrist rotator, 70mm and 143g). [13]

In addition to these mechanical features, the wrist also boasted a custom power management and signal communication setup. Power (2-cell battery) and raw EMG data (using CAN communication) lines were fed through the central axis of the rotator. Data lines were sent directly to the microcontroller, while power lines were fed through an inline fuse, reverse voltage protection, and over/under voltage protection. After this stage, voltage was boosted up to 12 V to power the Dynamixel actuator modules, bucked down to 5 V to power the electronics, and fed through as is to power the various terminal devices at a nominal voltage of 7.4 V. Signal communication for the terminal device was handled with a terminal device routing board, where the various data lines using analog and I2C communication protocols (depending on the terminal device) were routed with the input power/ground lines along the hinged frame using FPC cables. The wrist also contained an externally facing USB-C port for offloading device data from the on-board SD card and for updating device firmware directly to the microcontroller.

### C. Prosthesis Configuration.

**Fig. 2.**
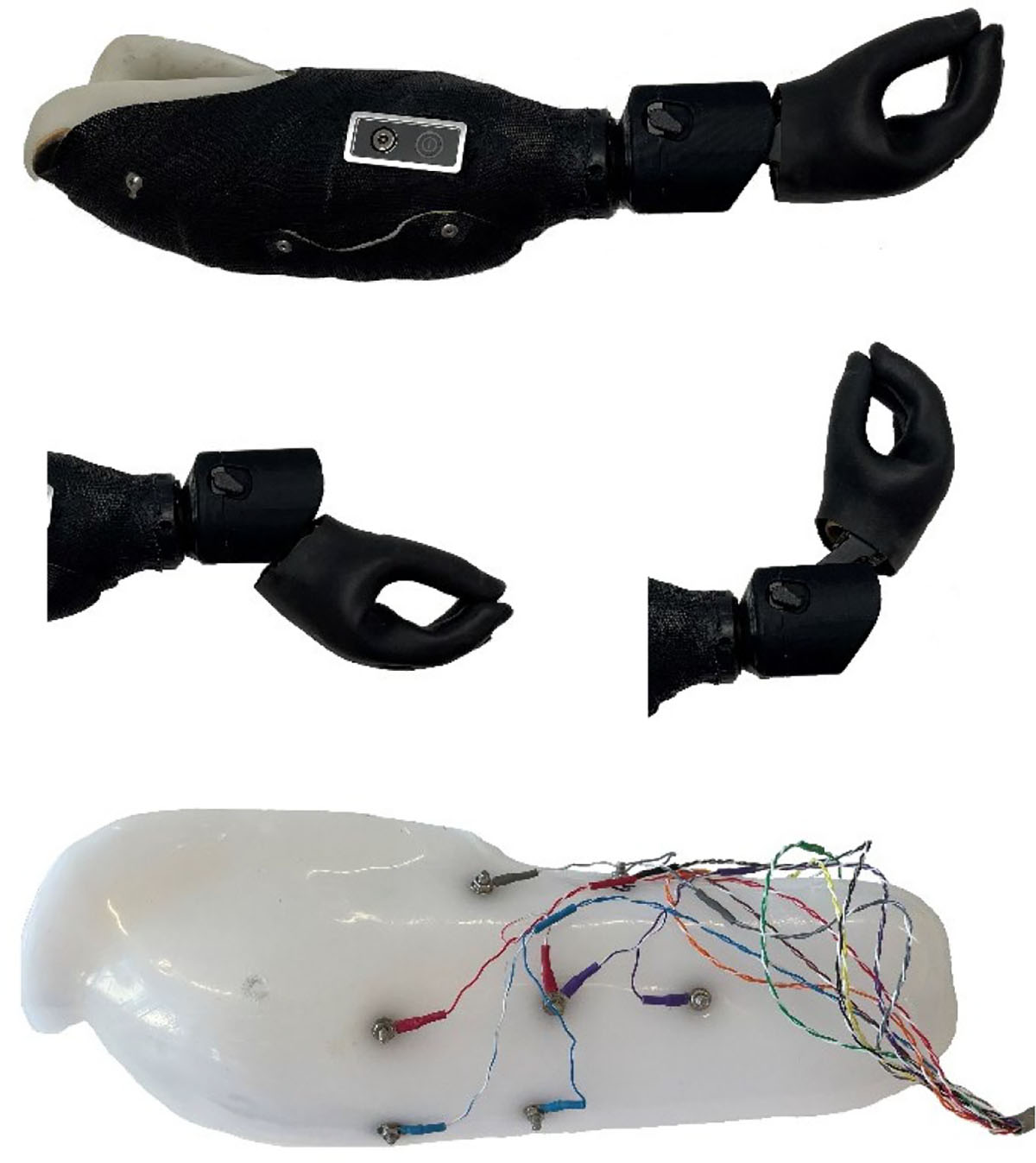
Photograph of the full prosthetic system with wrist in neutral position including the 2-DOF wrist and an Ottobock Transcarpal Hand DMC plus hand. (top). Wrist in full extension and flexion (middle). A flexible inner socket (bottom), showing an example of the distribution of the 8 bipolar EMG channels.

Participants were fit with the custom-designed 2-DOF prosthetic wrist capable of 351° rotation and 100.5° flexion/extension (58° flexion, 42.5° extension), integrated with an Ottobock Transcarpal Hand DMC plus hand (Fig 2). The components were mounted to a dual-layer socket: a flexible inner socket and a rigid fiberglass outer shell. Where possible, socket fabrication was based on duplication and modification of the participant’s home prosthesis to maintain familiarity and optimize comfort.

### D. Control System.

We implemented an 8-channel, pattern recognition–based EMG control system for prosthesis operation. EMG signals were amplified using a custom analog front-end built around the Texas Instruments ADS1299 instrumentation amplifier. The amplifier gain was set to 2, and a digital band-pass filter was applied with a passband of 20–300 Hz. Signals were sampled at 500 Hz.

All control code was implemented on the Pyboard D-Series SF6W and written in the Micropython language [14]. The pattern recognition system implemented was similar to the system described by Englehart and Hudgins [15]. Data were windowed into 200 ms analysis windows, and time-domain features were used to extract discriminatory information from the signals. This resulted in a 32 dimensional feature vector that was classified using a linear discriminant analysis-based classifier. Proportional control was achieved using the motion normalized proportional control approach described previously by Scheme et al [16]. Classification outputs were then processed using a velocity based smoothing ramp as described by Simon et al to attenuate any transient misclassifications [17]. Control decisions were made every 25ms which was fast enough to appear as a continuous control output to the end users.

Pattern recognition systems require calibration data. This was achieved through a combination of screen-guided training and prosthesis guided training approaches [18]. In screen guided training, a user follows the instructions of a mobile application to perform instructed movements, whereas in prosthesis guided training the user is instructed to mimic the movement of the prosthesis. In this study, screen guided contractions last 3 seconds, and each prosthesis movement was programmed to also last for approximately 3 seconds in order to move through its range of motion.

For the first condition, the system was calibrated to recognize five distinct motion classes: no movement, wrist pronation, wrist supination, hand open, and hand close. For the second condition, wrist flexion and wrist extension were added. All DOFs—wrist rotation, flexion/extension, and hand grasp—were calibrated using movement patterns identified with training from an occupational therapist. We did not require a minimum or maximum number of calibrations to train the pattern recognition system. Rather, we attempted to mimic the clinical delivery of the systems where the occupational therapist would instruct the participants on how to calibrate, and participants could choose to add to the classifier throughout the experiment. The flexion/extension unit was selectively disabled and held in a locked neutral position for Condition 1 (allowing rotation only) and enabled for Condition 2 (allowing rotation and flexion/extension). The same hand was used for both conditions.

### E. Study Protocol.

Participants underwent in-lab training with an occupational therapist before completing a series of functional assessments under both conditions. Conditions were not randomized and participants always started with the rotation-only configuration, in order to mirror typical clinical progression to a more complex system. [18], [19] Between test conditions, participants used the prosthesis at home when feasible; however, due to COVID-19-related constraints, not all participants completed home trials (Table I). Pre-home trial test results were used for consistency.

### F. Outcome Measures.

For each control condition, participants completed a brief warm-up period (10–15 minutes), followed by a series of standardized functional assessments [12]. These included the Box and Blocks Test (BBT; the number of blocks moved in one minute, repeated for three trials) [20], the Clothespin Relocation Test (CRT; the time required to move three clothespins from a horizontal to a vertical bar, repeated for three trials) [21], the Jebsen Taylor Hand Function Test (a timed test of simulated functional hand tasks representing 7 activities of daily living, one trial) [22], the Southampton Hand Assessment (SHAP; a timed test of grasp and manipulation across abstract and ADL-related objects, one trial) [23], the Activities Measure for Upper Limb Amputees (AM-ULA; a scored evaluation of predefined, simulated activities of daily living, one trial) [24], and the Assessment of Capacity for Myoelectric Control (ACMC; a clinician-rated measure of myoelectric control during a bimanual functional task, one trial) [25]. Testing was typically completed within 2–3 hours, with rest breaks provided as needed. Each device condition was evaluated on a separate day.

### G. Statistical Analysis.

The data consisted of measurements from 5 participants completing 6 different outcomes under two conditions: without wrist flexion (noWF) and with wrist flexion (WF). Paired t-tests were conducted using the ttest_rel function from the Python scipy.stats library for each outcome. A significance level was defined as a p-value greater than 0.05.

## III. Results

**Fig. 4.**
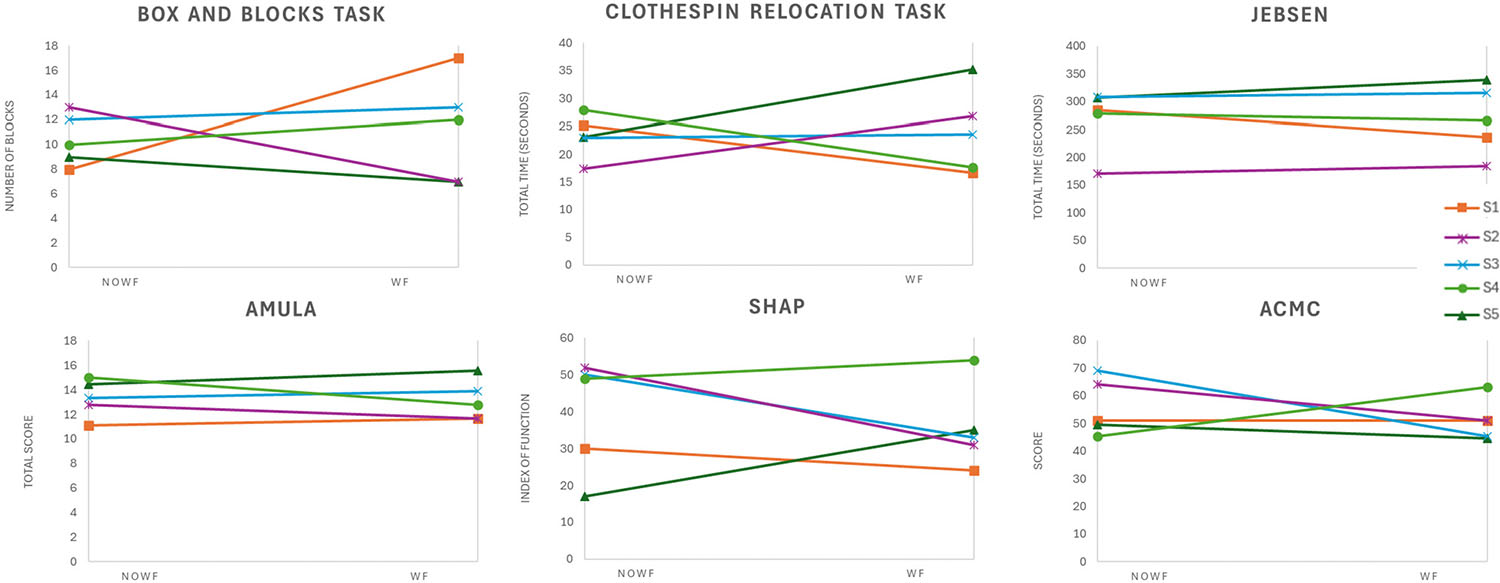
Outcome results for the five participants. Top row, rom left to right: Box and Blocks (number of blocks moved in 1 minute), Clothespin Relocation Task (time to move three clothespins), Jebsen Taylor Test of Hand Function (total time for all seven tasks). Bottom row, from left to right: AM-ULA (total score), SHAP (Index of Function), ACMC (total score). Improved performance is indicated by a higher score for the Box and Blocks, AM-ULA, SHAP, and ACMC, and a lower score for the Clothespin Relocation Task and Jebsen.

Though some individuals did improve functional outcomes with the addition of wrist flexion, others had difficulty with the control of a more complex system, resulting in lower outcome scores. Individual outcomes comparing the two conditions are graphically presented in Figure 4. Overall, none of the group mean outcomes showed statistically significant differences between the noWF and WF conditions based on paired t-tests, as indicated by p-values greater than 0.05.

For the Box and Blocks Test, the average blocks moved per minute was 10.33 ± 3.18 (mean + standard deviation) without wrist flexion as compared to 10.87 ± 4.21 with wrist flexion [t-statistic = −0.367, p-value = 0.719].

For the Clothespin Relocation Task, the group results time to move the 3 clothespins for noWF and WF were 23.29 ± 5.05 and 23.98 ± 8.07, respectively [t-statistic = −0.271, p-value = 0.790].

Mean results for the Jebsen Taylor Test of Hand Function (maximum possible score 840 s) were 269.85 ± 56.72 for noWF and 268.40 ± 61.79 with WF [t-statistic = 0.106, p-value = 0.920].

SHAP index of function (possible range 0–100) mean group results for noWF and WF were 39.60 ± 15.44 and 35.40 ± 11.19 [t-statistic = 0.586, p-value = 0.589].

Results of the AM-ULA (possible range 0–40) means were 13.33 ± 1.52 with noWF and 13.11 ± 1.65 with WF. [t-statistic = 0.356, p-value = 0.740]

For the ACMC (possible range 0–100) the group average for both conditions was “generally capable” with a score of 55.82 ± 10.16 for noWF and 51.06 ± 1.48 with WF [t-statistic = 0.691, p-value = 0.528].

## IV. Discussion

In this study, we evaluated whether the addition of active wrist flexion and extension to a prosthesis with powered wrist rotation would enhance functional task performance for individuals with transradial limb loss. Across a battery of validated upper-limb outcome measures, group-level results revealed modest differences between the wrist rotation-only (noWF) and wrist rotation plus flexion (WF) conditions. These findings suggest that, for the tasks evaluated, the inclusion of a second powered degree of freedom at the wrist does not significantly increase control complexity to compromise control performance but also may not yield immediate or measurable performance gains in short-term laboratory settings.

Despite expectations that enhanced wrist mobility would reduce compensatory movements and improve task efficiency, the impact of the added mass and length (current 2DOF wrist: 342 g and 94.2 mm, compared to a commercial Motion Control wrist rotator: 143g and 70mm [13]) when combined with increased control complexity of the 2-DOF configuration may have tempered potential benefits. However, it is also beneficial to note that, overall, task performance was also not negatively impacted by a more complex system. Tasks such as the Jebsen Taylor and Clothespin Relocation, which emphasize speed and coordination, may have been sensitive to even subtle increases in prosthetic mass or control effort.

Importantly, this study was conducted in a constrained timeframe, and most participants were unable to complete an extended home-use phase with either condition, limiting analysis to the pre-home trial outcomes. Given previous literature demonstrating learning effects and adaptation with novel prosthetic components, it is plausible that longer-term use could yield different results [9], [26]. It is also possible that condition order (rotation-only first) could have provided a learning advantage to the second condition (rotation + flexion/extension), potentially impacting performance. Though this follows clinical training recommendations, future studies may consider the length of the familiarization periods before testing, wash-in/wash-out phases, or direct randomization. The small sample size further limits the ability to detect statistically significant differences, although individual-level trends suggest that some participants may have benefitted from the added wrist functionality.

While statistical significance was not observed, it is notable that function did not degrade with the addition of powered wrist flexion/extension. This finding supports the feasibility of integrating additional degrees of freedom without compromising core prosthetic performance. From a clinical perspective, this may be relevant in scenarios where specific tasks, such as midline reach, self-care, or object manipulation at varying heights, require enhanced wrist maneuverability, even if not captured by standard outcome metrics.

It is possible that continued development and improvement to the control may allow for easier use of this additional degree-of-freedom. For this study, 8 channels of EMG were used as input to an LDA pattern recognition classifier. This was based on past work showing that 6 optimally placed channels of EMG only reduced classification accuracy from 93.1% to 91.5% for 6 movements when compared to using 12 channels of EMG [27]. However, more recent work has shown that increasing the number of EMG channels may facilitate control and improve outcomes and user perception [28]. For this more complex system including additional electrodes may have translated to improved control. Additional work on more advanced machine learning control systems may also increase performance for heavier and more complex systems [29]. Finally, although we attempted to employ parameters used in commercially available pattern recognition control systems, the algorithm was implemented on a Pyboard D-Series SF6W off-the-shelf microcontroller. Any proprietary advances in control or user training developed by commercial manufacturers were not included in our study.

Future studies should explore longer-duration home use, assess compensatory motion via kinematic analysis, and incorporate user preference and qualitative feedback to better understand the perceived value of added wrist function. It may also be beneficial to record classification decisions throughout each trial to better document usage. Additionally, further optimization of control algorithms, training protocols, and device size and design may be necessary to fully realize the potential benefits of multi-DOF prosthetic wrists. However, in spite of our study limitations we were able to show that a two-degree powered wrist device could be used by individuals with only a modest amount of training, and any improvements made would presumably only make such a system more functional.

## Figures and Tables

**Fig. 3.**
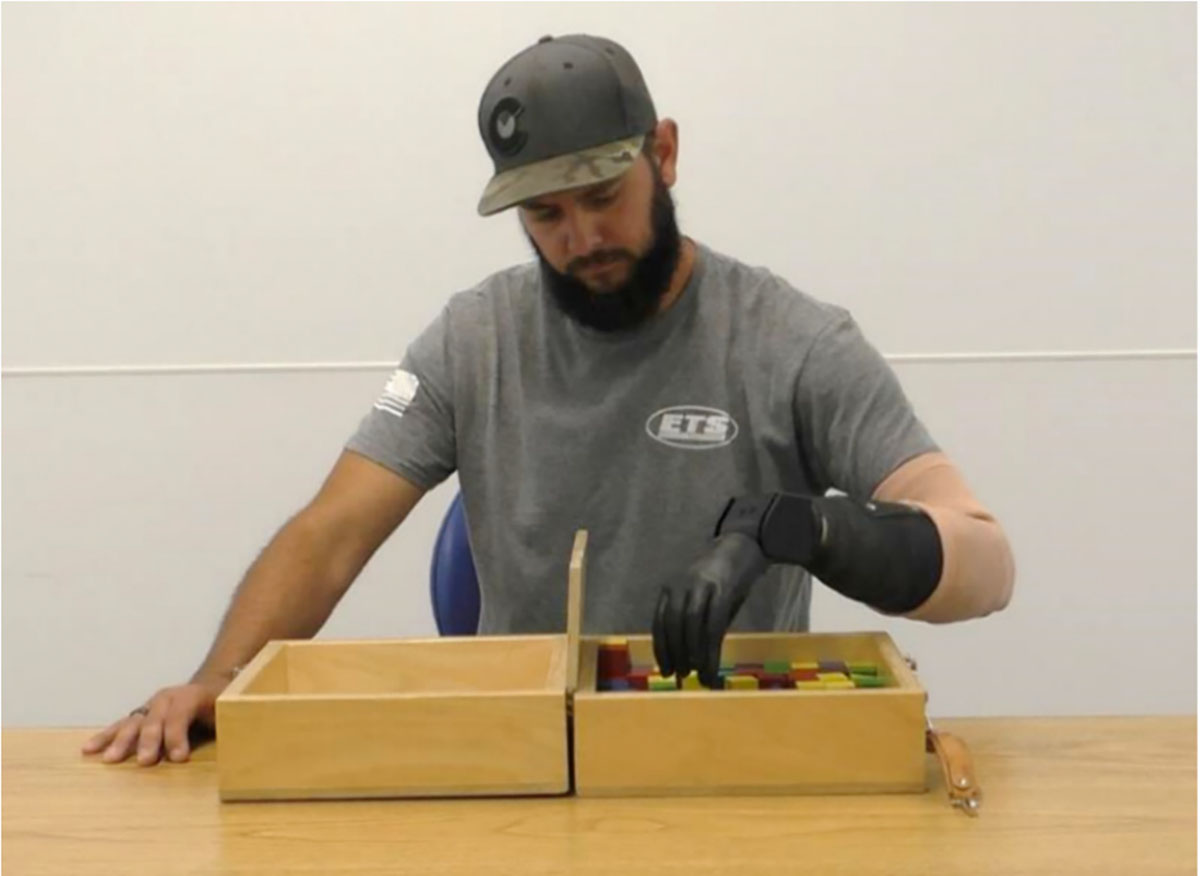
Photograph of a participant wearing the configured prosthesis, with wrist flexion enabled, to perform the Box and Blocks experiment.

**TABLE I:** Participant Demographics

	Side	TMR	Primary home device at time of participation	Age	Home trial noWF/WF
S1	R	N	Myo with 2-site control: Taska, passive wrist	55	Y/Y
S2	L	Y	None: Abandon use	34	Y/N
S3	L	N	Myo with 2-site control: Bebionic, passive wrist	28	Y/Y
S4	R	N	Body powered: TRS Jaws	40	N/N
S5	L	Y	Equal use of Body powered: 5x hook & Myo with Pattern recognition control: Bebionic hand and passive wrist	32	Y/N
